# Supplementary material for: Triage Strategies Based on C-Reactive Protein Levels and SARS-CoV-2 Tests among Individuals Referred with Suspected COVID-19: A Prospective Cohort Study
Source: J Clin Med. 2021 Dec 30;11(1):201. doi: 10.3390/jcm11010201 (PMC8745759; doi:10.3390/jcm11010201)
Supplement: Supplementary file 1 [file jcm-11-00201-s001.zip › Supplementary Material File S1 Original protocol.pdf]

Project:

Triage strategies based on C-reactive protein levels among individuals referred with suspected COVID-19: A concise protocol for a prospective cohort study

By: Henning Bliddal, Marius Henriksen, Lennart Friis-Hansen, Lars E. Kristensen, Charlotte Rahbek, Jens Rasmussen, Robin Christensen. From Bispebjerg Frederiksberg Hospital, Depts of Acute medicin, Clinical Chemistry and the Parker Institute.

Introduction

Coronavirus disease 2019 (COVID-19) is caused by the newly discovered coronavirus, SARS-CoV-2. No effective prophylactic or post-exposure therapy is currently available. The Covid-19 pandemic has led to severe shortages of many essential goods and services, including tests for the virus. The worsening of the virus infection from onset of symptoms of COVID-19 to development of acute respiratory distress syndrome ARDS has been reported to be associated with a concomitant increase in C-Reactive Protein (CRP) {Li LQ, et al. J Med Virol 2020}. The present study deals with less affected individuals, with the aim of predicting cases, which might develop into ARDS.

Rationale for this study

At Bispebjerg and Frederiksberg Hospital, a clinical prescreening of individuals is performed at a unit at the entry point of the Department of Acute Medicine. This unit named, *Corona-Check-Point* (CCP), is staffed with qualified nurses and doctors, and the triage whether or not to admit to the Department is based on the history and clinical picture, while test results for virus are only available after 6-8 hours. At the CCP, about 150 persons are seen each day, mainly during day time, and less than 10 per cent are admitted. The majority of patients seeking diagnostic advise based on their symptoms following the necessary visitation will be informed that they should go back home and contact a doctor in case of deterioration.

Aim

To examine whether a high level of CRP at the onset of COVID-19 symptoms, predicts hospitalization and possibly later development of ARDS.

Objective

To compare the prognostic value of having a high CRP, relative to individuals with a low CRP level, on risk of developing ARDS among patients diagnosed with COVID-19 at baseline.

Perspective

Considerable resources are used for testing for COVID-19. If a simpler analysis, e.g. CRP, could take over in a triage situation, more tests at a much lower budget would be feasible. As for relevant submissions of COVID-19 infected, a coming treatment strategy would prosper from earlier diagnosis of the worst cases to come.

Methods

We will carry out this single-center study using prospective data from the CCP unit and Department of Acute Medicine, Bispebjerg Frederiksberg Hospital, starting in April 2020, for one month, and analyzed in June 2020. We will include adults presenting at the Dept of Acute Medicine with a suspicion of COVID-19, whether referred from general practice or by self-appearance. Exclusion criteria will be examination at the CCP unit or hospitalization for the same diagnosis within the past 28 days. Participants will be registered in the Official filing system (Sundhedsplatformen, SP) and followed up by file for one month. Blood samples will be drawn by a team of medical students in the hours 8-24. The project will be submitted to clinicalTrials.gov.

Database and registry

Data are extracted from SP, including CRP level, both at the CCP and possible later measurements, vital parameters/measures, COVID-19 test results, hospitalization including length of stay and intensive care, assisted respiratory treatment, death.

Biobank, Definitions and measurement of C-reactive protein levels

The test for CRP will be performed as part of the routine work at the Bispebjerg Hospital. A sample will be stored for later analysis according to the Biobank-project in the Region (Iversen et al. P-2020-262). In COVID-19 infected persons, no exact data are published, however, we shall à priori use a dichotomous cut-off by the median value.

Sample size and power considerations

All available data on the database will be used to maximize the power and generalizability of the results. Our sample size calculation is based on our primary objective (i.e., to determine if high levels of CRP has additional predictive value beyond clinical variables predicting who will develop ARDS within 30 days). We expect a to enroll 3000 individuals with suspected COVID-19, If our event rate (developing ARDS) is 5% on average, a total sample size of 3000 individuals (i.e. 1500 with high CRP [above the median]) we will have a good statistical power (>96%) to detect a difference between proportions developing ARDS 3% points (6.5% and 3.5%, respectively).

CV for: Henning Bliddal, MD, DMSci, Professor of Rheumatology

Henning Bliddal is a specialist in rheumatology. Since 1997 he has been Leader and Professor of Research at the Parker Institute, a clinical research unit of Rheumatology, Copenhagen University Hospital, Bispebjerg and Frederiksberg, Denmark.

HB's research projects have over the last years concentrated on a. broad range of rheumatological diseases.

HB is an experienced leader of research projects and is currently coordinator of the HORIZON 2020 project, ELECTOR.

HB has supervised numerous medical students and physicians, including 32 PhD students. He has extensive teaching experience and is a regular contributor and guest speaker at national and international congresses.

HB has contributed to textbooks, and has published more than 350 papers (dec. 2019) in international medical journals with peer review. For details please refer to PubMed and Embase.

H-index 64, citations >16000

Dr. H. Bliddal, The Parker Institute, Bispebjerg and Frederiksberg Hospital, Denmark.

Nordre Fasanvej 57 {Vej 8, Indgang 19}, DK-2000 Copenhagen F, Denmark. e-mail: Henning.Bliddal@regionh.dk

Tel: +45 3816 4151/Fax: +45 3816 4159/ORCID iD: https://orcid.org/0000-0002-7951-1668

Cph March 31, 2020

H. Bliddal

Budget:

Budget		
Start ASAP		
**Salaries**		År 1
Marius Henriksen	Trial Coordinator	70.000,00
Robin Christensen	Biostat.	50.000,00
Christian Cato Holm	Databasemanager	50.000,00
Henning Bliddal	PI Covered by BFH	0,00
Medical Students (FADL)	24 Hours in 30 days	90.000,00
Secretary	2 months	80.000,00
		340.000,00
Blood tests	Covered by BFH	
Publication costs		15.000,00
		15.000,00
		355.000,00
Administrative Fee 5%		17.750,00
In total:		372.750,00
