# Supplementary material for: Triage Strategies Based on C-Reactive Protein Levels and SARS-CoV-2 Tests among Individuals Referred with Suspected COVID-19: A Prospective Cohort Study
Source: J Clin Med. 2021 Dec 30;11(1):201. doi: 10.3390/jcm11010201 (PMC8745759; doi:10.3390/jcm11010201)
Supplement: Supplementary file 1 [file jcm-11-00201-s001.zip › Supplementary Material File S2 Statistical Analysis Plan_1.2021April13.pdf]

- **Statistical Analysis Plan** -

Triage strategies based on C-reactive protein levels and COVID-19 positivity among individuals referred with suspected COVID-19

A concise statistical analysis plan for a prospective cohort study

v. 1. 2021-04-13

Erika Olivia Boyesen[1], Ida Maria Balsby[1], Marius Henriksen[1], Robin Christensen[1], Jens Rasmussen[2], Finn Erland Nielsen[2], Lennart Friis Hansen[3], Susanne Dam Nielsen[4], Rebekka Faber Thudium[4], Celeste Porsberg[5], Lars Erik Kristensen[1], Henning Bliddal[1].

1: The Parker Institute, Bispebjerg and Frederiksberg Hospital, Copenhagen, Denmark

2: Department of Acute Medicine, Bispebjerg-Frederiksberg Hospital

3: Department of Clinical Biochemistry, Bispebjerg-Frederiksberg Hospital

4: Department of Infectious Diseases 8632, Rigshospitalet

5: Department of Pulmonary Medicine, Bispebjerg-Frederiksberg Hospital

INTRODUCTION

Coronavirus disease 2019 (COVID-19) is caused by severe acute respiratory syndrome coronavirus 2, SARS-CoV-2 (Pulia *et al.*, 2020). The virus was initially discovered in Wuhan, China in December 2019. In March 2020, The World Health Organization declared a COVID-19 pandemic and since then, the infection rate has increased exponentially worldwide (Chilimuri *et al.*, 2020). As of March 2021, the coronavirus disease has resulted in over 120,000,000 confirmed cases and more than 2,600,000 deaths globally (WHO, 2021). In Denmark, March 18th 2021, there were 223,400 infected patients and 2,397 deaths in total (sst.dk, 2021). COVID-19 can result in the development of acute respiratory distress syndrome (ARDS), which is considered a high mortality disease (Zhang *et al.*, 2020). The pathological picture of ARDS includes fibrin deposition, diffuse alveolar damage with hyalin membrane formation, perivascular T cell infiltration etc. (Asselah *et al.*, 2021).

The worsening of the virus infection from onset of symptoms to development of ARDS has been reported to be associated with a concomitant increase in C-Reactive Protein, CRP (Keddie *et al.*, 2020). Furthermore, the pro-inflammatory cytokine interleukin-6, IL-6, has shown prognostic value of in-hospital deaths (Liu *et al.*, 2020). Since the production of CRP in the liver is driven by IL-6 (Keddie *et al.*, 2020), it supports the rationale behind investigating the role of isolated CRP in the early stage in predicting the course of the disease. The present study deals with non-hospitalized patients with varying severity of the COVID-19 infection. Based on one simple biomarker (CRP), it is our aim to possibly predict the development of hospitalization and/or ARDS among COVID-19 patients with a CRP above the clinical threshold of 10 mg/L. There is potential for an effective triage strategy if a CRP result can separate COVID-19 patients with an increased risk of getting admitted from patients who are safe to be home discharged.

Rationale and aim for this study

At Bispebjerg Frederiksberg Hospital and at Rigshospitalet in Copenhagen, Denmark, a clinical prescreening of individuals was performed at a unit, *Corona-Check-Point* (CCP), at the entry point of the Department of Acute Medicine and Department of Infectious Diseases. The CCPs were staffed with qualified nurses, medical students and doctors who collected blood samples/COVID-19 tests and consulted with the patients. The triage, whether to admit the patients or not, was based on the

history and clinical picture, while test results for CRP and virus were not available until after 6-8 hours.

Current recommendations for management of COVID-19 include large-scale tests for virus. Such tests reveal whether an individual is infected with the virus. However, the demonstration of virus *per se* has – to our knowledge - no prognostic value for the ensuing course of the COVID-19 disease. The number of publications describing possible treatment strategies are increasing (Stasi *et al.*, 2020). More studies show that CRP and other biochemical parameters are increased in hospitalized COVID-19 patients. However, evident effective triage strategies of pre-hospitalized COVID-19 patients are still to be clarified.

This trial asks a previously unaddressed and important clinical question about triage strategies and management of the COVID-19 infection in the early stages. We hypothesize that the value of a CRP measurement for triage of patients initially presenting (at baseline) with light signs of a COVID-19 infection will be clinically valuable. Regarding decision making for which patients are to be hospitalized due to risk of developing more severe symptoms, this study addresses the question whether triage may be performed with the aid of a simple CRP measurement.

OBJECTIVES

Our primary objective is to compare the prognostic value of CRP (above the reference range), a positive COVID-19 test, the combination of these, and the observed risk of being hospitalized (during 28 days of observation) among patients referred with suspected COVID-19 at baseline.

Secondary objectives include comparing the prognostic value of CRP, the result of a COVID-19 test, the combination of these, and the observed risk of needing oxygen treatment during hospitalization, transfer to the Intensive Care Unit, and mortality (during the 28 days of observation) among patients referred with suspected COVID-19 at baseline.

METHODS

Study design

This prospective cohort study was designed as a multicenter study, carried out using prospective data from the CCP units at the Department of Acute Medicine, Bispebjerg Frederiksberg Hospital and from the Department of Infectious Diseases, Rigshospitalet, starting in May 2020. The project objective and outline were submitted to ClinicalTrials.gov NCT04373798. The study was approved by the Danish Data Protection Agency.

As illustrated in **Figure S1**, the study design and setting will be visualized in the "study profile" illustrated in a STROBE flow diagram (Vandenbroucke *et al.*, 2007).

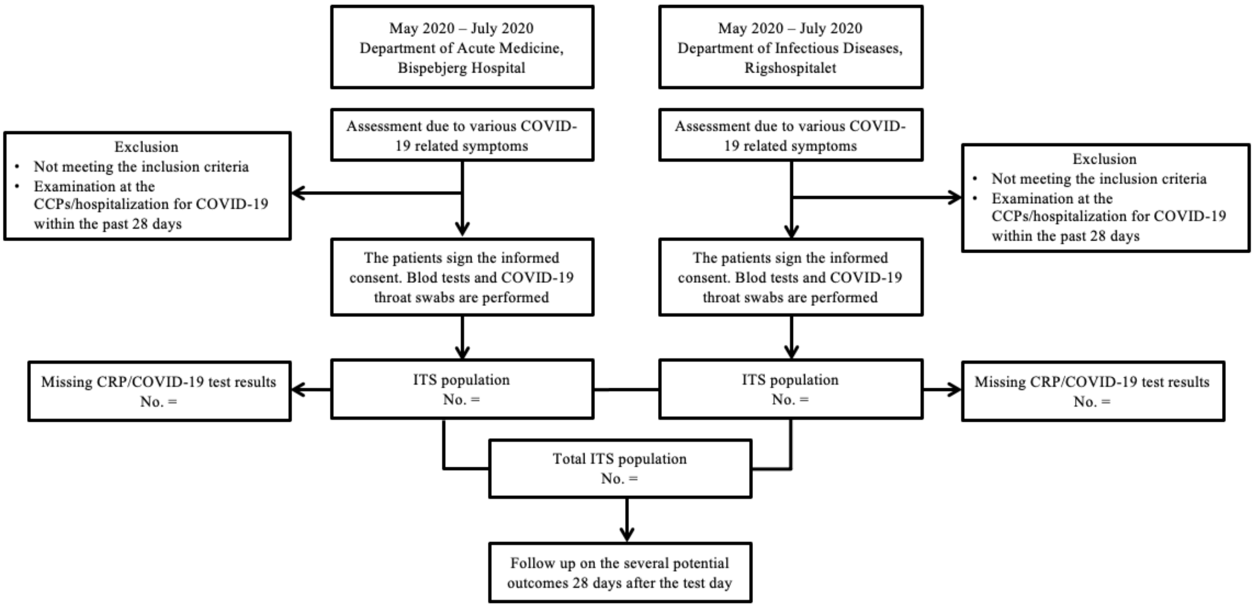

Figure S1. We will report the flow of participants through each stage of the prospective cohort study.

Setting and participants

Individuals (aged 18 and above) presenting during daytime (8 am to 5 pm) at the Department of Acute Medicine and the Department of Infectious Diseases, with a suspicion of COVID-19, whether referred from general practice or by self-appearance, were asked to participate in the prospective data

collection. All participants presented with COVID-19 related symptoms such as fever, muscle soreness, headache, coughing etc.

Exclusion criteria were examination at one of the CCP units or hospitalization for the same diagnosis within the past 28 days. Participants were registered in the Official filing system (EPIC/Sundhedsplatformen, SP) and followed up by file for one month (28 days). Blood samples were drawn by a team of medical students.

The primary analyses will be based on the Intention to Survey (ITS) population. The ITS principle will assert the effect of the initial exposure (that is, the result of the COVID-19 and CRP level at enrolment). Our ITS population will exclusively be participants who signed the informed consent and had both the CRP assessed and the result of COVID-19 available at baseline. Accordingly, participants will be eligible for "allocation" to a prognostic group (e.g., High CRP and Low CRP). They will be followed up, assessed and analyzed as members of that group, regardless of their clinical history from that point on (e.g., independent of withdrawals and cross-over phenomena).

Data sources/measurements

Data will be extracted from the EPIC (Medical Records), including the CRP level, both at the time of appearance at the CCPs and at possible later measurements, COVID-19 test results, hospitalization (adjusted to COVID-19 related hospitalizations). The data extraction includes length of stay and intensive care, assisted respiratory treatments, deaths, demographic information (sex and age). The test for CRP was performed as part of the routine work at Rigshospitalet and Bispebjerg Frederiksberg Hospital.

Quantitative variables

Initially in the statistical modelling, patients with a CRP above the clinical threshold (10 mg/L) will be considered as individuals with a "*high* CRP" (unlike those below the threshold; "*low* CRP"). Secondarily, we will explore the association between CRP and hospitalization using various other thresholds defined by data-driven categories that relate to hospitalization within 28 days from presenting at the CCPs with a suspicion of COVID-19. A Receiver Operating Characteristic (ROC)

curve will be used to graphically show the connection/trade-off between clinical sensitivity and specificity for every possible cut-off for the baseline CRP levels.

Diagnostic Test(s) for SARS-CoV-2:

In this study, samples were collected by oropharyngeal swabs using the UTM swab set (COPAN, Brescia, Italy) followed by later laboratory RT-PCR analyzes detecting viral nucleic acids with the Roche FLOW System (Roche, Basel, Switzerland). This diagnostic test has a good efficiency and is nationally being performed thousands of times a day (i.e. in Denmark). The analysis method expresses a testing sensitivity of 95%, which is one of the reasons RT-PCR is the leading diagnostic test for accurate and rapid detection of the COVID-19 infection (D'Cruz, Currier and Sampson, 2020).

Diagnostic Kit measuring CRP:

CRP was measured in heparin plasma using the c702 analyzer (Roche Diagnostics, Mannheim, Germany). The CV% was 7.5% at 7.6 mg/L.

Statistical methods

Accurate prognostic information is vitally important for patients and physicians to make optimal decisions. Clinicians involved with triage strategies while examining individuals with possible COVID-19, are interested in clinical prediction; i.e. they want to know (with some degree of uncertainty) which individuals will get a severe disease course (such as being hospitalized, needing oxygen, being transferred to the ICU, or maybe even dying), and which will not. Logistic regression analysis models will be used for this. All *P* values and 95% confidence intervals will be two sided. We will not apply explicit adjustments for multiplicity, rather we will analyze the key secondary outcomes in the following prioritized order:

- Needing oxygen (within 28 days)
- Being transferred to the ICU (within 28 days)

- Mortality (within 28 days)

In randomized controlled trials (RCTs), randomization would ensure that, on average, treated subjects (say those exposed; high CRP level) will not differ systematically from untreated subjects (those unexposed; high CRP level) in both measured and unmeasured baseline characteristics. In contrast, a non-randomized study like the present one, looking at the impact of COVID-19 and the CRP level on outcomes, can be subject to confounding bias in which either 'High CRP' or COVID-19 positive individuals differ systematically from 'Low CRP' or COVID-19 negative (Sterne *et al.*, 2016).

As illustrated in **Table S1**, we will initially analyze the COVID-19 (positive vs. negative) test status and CRP level at the time of enrolment. For all the measures collected at baseline we will compare the means of baseline CRP levels and CRP above and below the threshold (10 mg/L) with COVID-19 positive and COVID-19 negative tests in the sample. Since all the outcome measures (dependent variables) are dichotomous we will use logistic regression.

Table S1. Participant characteristics at baseline stratified according to subsequent COVID-19 positivity

**Characteristics**	**BFH**		**RH**		**Total**	
	COVID-19 pos.	COVID-19 neg.	COVID-19 pos.	COVID-19 neg.	COVID-19 pos.	COVID-19 neg.
	n =	*n* =	*n* =	*n* =	*n* =	*n* =
Males, no. (%)						
Age, years						
CRP level, mg/L						
CRP < 1 mg/L, no. (%)						
CRP between < 1 mg/L and ≤ 10 mg/L, no. (%)						
CRP > 10 mg/L, n (%)						

In **Figure S2A**, we aim to illustrate the association between the risk of patients being admitted as the dependent variable (y) while the baseline CRP level will be used as the independent variable (x).

In **Figure S2B**, we will use the receiver operating characteristic (ROC) curve to illustrate sensitivity as a function of (100% - specificity), determining a CRP threshold for hospitalization among COVID-19 positive and negative individuals. The true positive rate (TPR) is plotted against the false positive rate (FPR) related to possible cut off values of CRP.

Figure S2A. Association between the risk of patients being admitted vs baseline CRP.

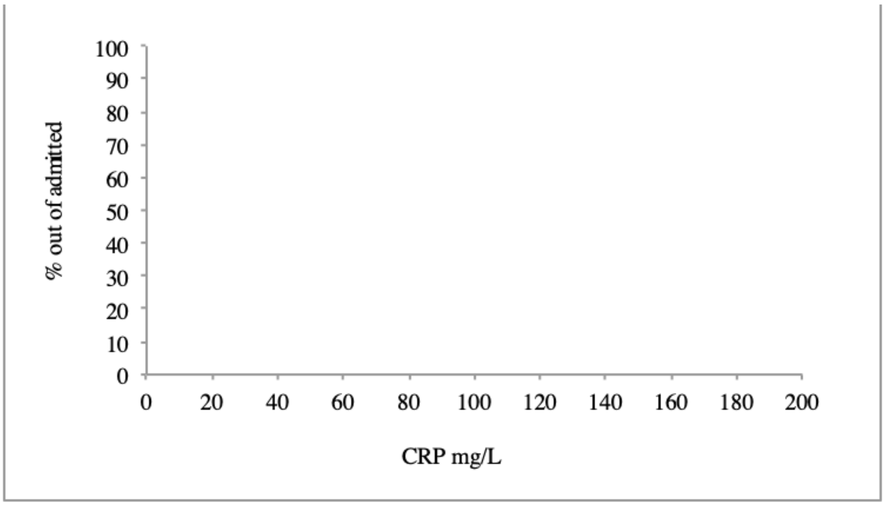

Figure S2B. ROC curve with sensitivity as a function of (100% - specificity). CRP level and hospitalization

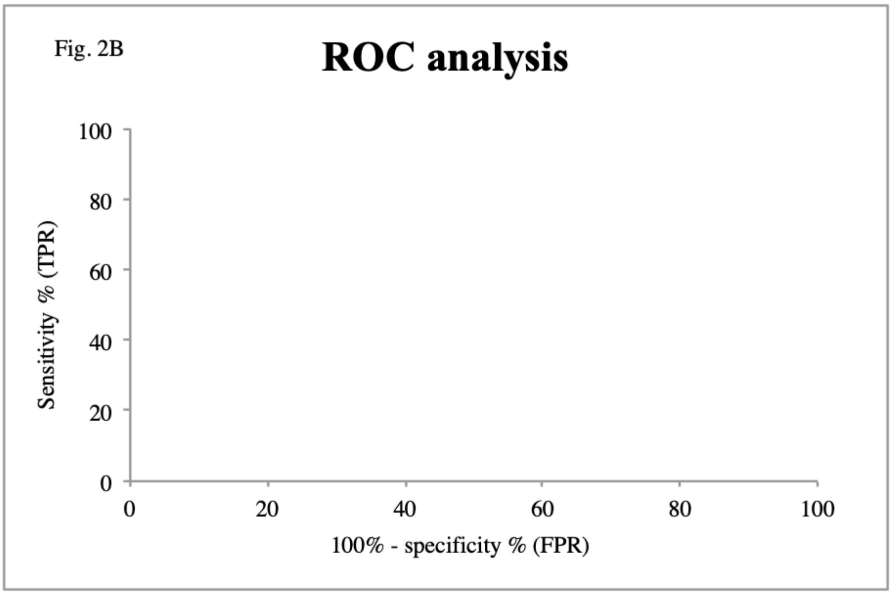

In **Table S2A**, we will illustrate the baseline CRP results (respectively below and above the 10 mg/L threshold) as the independent variables in relation to the dependent variables, including positive COVID-19 test results, admission, oxygen treatment, transfer to ICU and death. Using univariable logistic regression models and deriving (unadjusted) odds ratios with 95% confidence intervals, and P-values, we will examine the associations between individual baseline characteristics of participants (CRP level) and development of hospitalization etc. within 28 days (from baseline).

Table S2B presents the association between a COVID-19 result (positive vs. negative) as the independent variable and the several outcomes as well as the mean CRP level (mg/L). This table reveals if the mean baseline CRP in general is higher among the group of COVID-19 positive participants compared to the COVID-19 negative participants. Furthermore, it will show if there is a greater risk of hospitalization, oxygen treatment, transfer to ICU/ventilator or even death when infected with COVID-19 (independently of the CRP result). Using univariate logistic regression models and deriving (unadjusted) odds ratios with 95% confidence intervals and P-values, the associations between the COVID-19 results and hospitalization etc. within 28 days (from baseline) will be investigated.

Table S2A. Univariate logistic regression: Odds Ratios, 95% Confidence Intervals, and P-values, for all outcomes (crude analyses). CRP category level as independent variables compared to COVID-19 positivity and the various outcomes (dependent variables)

**Outcomes**	**CRP ≤ 10 mg/L** *n =*	**CRP > 10 mg/L** *n =*	**Standardized Difference**	**Odds Ratio (OR)**	**95% Confidence interval**	**P-value**
COVID-19 positive, n (%)						
Admitted, n (%)						
Oxygen treatment, n (%)						
Transfer to ICU/ventilator, n (%)						
Transfer to ICU, n (%)						
Ventilator, n (%)						
Death, n (%)						

Table S2B. Univariate logistic regression: Odds Ratios, 95% Confidence Intervals, and P-values, for all outcomes (crude analyses). COVID-19 results (positive vs. negative) as independent variables in relation to mean CRP and various outcomes (dependent variables).

Outcomes	COVID-19 positive<br><br>*n* =	COVID-19 negative<br><br>*n* =	Standardized<br><br>Difference	Odds Ratio (OR)	95% Confidence interval	P-value
CRP, mg/L						
Admitted, n (%)						
Oxygen treatment, n (%)						
Transfer to ICU/ventilator, n (%)						
Transfer to ICU, n (%)						
Ventilator, n (%)						
Death, n (%)						

Bias and Adjustment for possible confounding variables

The challenge with observational data is that group structures (e.g. COVID-19 positivity: y/n and High-CRP: y/n) are not applied randomly, potentially leading to biased inference due to unbalanced (confounding) variables. Improved confounding variable balance between the exposed (i.e. High-CRP [X_E]) and unexposed (i.e. Low-CRP [X_{UE}]) groups can be achieved by matching observations from each group based on the propensity score, which in this case would be the probability that a patient was enrolled with a CRP-value above the determined threshold (X_E) given the observed covariates. Propensity score methods attempt to correct for the assignment mechanism by finding unexposed units similar to exposed units $P(Y_0| X_E) \approx P(Y_0| X_{UE})$.

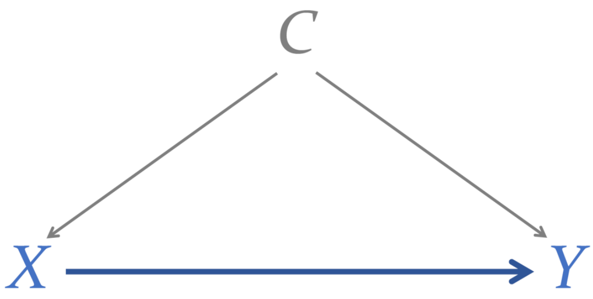

In order to specify possible propensity scores, we will use the following pragmatic definition of what makes a confounding variable (C):

- The Covariate (C) is an ancestor (cause) of the outcome (*Y*)
- The Covariate (C) probably causes the exposure (*X*; e.g. group)
- The Covariate (C) is ***not*** a descendant (effect) of the exposure (*X*) or outcome (*Y*)

From the data-driven process of developing the propensity score to each participant, i.e., the likelihood of having a High CRP level at baseline given that patient's status on other measured prognostic factors, we will conduct a logistic regression analysis adjusting for the data-driven propensity score. By this method we will include a few prognostic factors (as well as the result of the COVID-19 test), and thus transparently demonstrate whether the CRP level has an impact on hospitalization etc., while adjusting the propensity score as well as the result of the COVID-19 test.

Analysis population and handling of missing data

From the available data corresponding to the ITS population, missing (dependent outcome) data will be assumed '*Missing At Random*' (MAR) and will consequently be handled using the multiple imputation techniques (since we do not have any repeated measurements to include in a mixed effects model). Multiple imputation (MI) provides a useful strategy for dealing with data sets with missing values. Instead of filling in a single value for each missing value, the multiple imputation procedure replaces each missing value with a set of plausible or extreme values that represent the uncertainty about the right value to impute. Nonresponse in a sample survey like this will be handled by replacing each missing value with multiple imputations; five imputations will be applied and results from these datasets will be combined using Rubin's Rules. These multiple imputed data sets will then be analyzed by using standard procedures for complete data and combining the results from these analyses. We anticipate that this results in valid statistical inferences that properly reflect the uncertainty due to missing values (i.e. dependent outcomes).

FUNDING

This research received funding from Lundbeck Foundation (J.nr R349-2020-732). The Parker Institute, Bispebjerg Frederiksberg Hospital is supported by a core grant from the Oak Foundation (OCAY-18-774-OFIL).

REFERENCES

[1] Asselah, T. *et al.* (2021) ‘COVID-19: Discovery, diagnostics and drug development’, *Journal of Hepatology*, 74(1), pp. 168–184. doi: 10.1016/j.jhep.2020.09.031.

[2] Chilimuri, S. *et al.* (2020) ‘Predictors of mortality in adults admitted with COVID-19: Retrospective cohort study from New York City’, *Western Journal of Emergency Medicine*, 21(4), pp. 779–784. doi: 10.5811/westjem.2020.6.47919.

[3] D’Cruz, R. J., Currier, A. W. and Sampson, V. B. (2020) ‘Laboratory Testing Methods for Novel Severe Acute Respiratory Syndrome-Coronavirus-2 (SARS-CoV-2)’, *Frontiers in Cell and Developmental Biology*, 8(June), pp. 1–11. doi: 10.3389/fcell.2020.00468.

[4] Keddie, S. *et al.* (2020) ‘Laboratory biomarkers associated with COVID-19 severity and management’, *Clinical Immunology*, 221(August 2020), p. 108614. doi: 10.1016/j.clim.2020.108614.

[5] Liu, Y.-P. *et al.* (2020) ‘Combined use of the neutrophil-to-lymphocyte ratio and CRP to predict 7-day disease severity in 84 hospitalized patients with COVID-19 pneumonia: a retrospective cohort study’, *Annals of Translational Medicine*, 8(10), pp. 635–635. doi: 10.21037/atm-20-2372.

[6] Pulia, M. S. *et al.* (2020) ‘Multi-tiered screening and diagnosis strategy for COVID-19: a model for sustainable testing capacity in response to pandemic’, *Annals of Medicine*, 52(5), pp. 207–214. doi: 10.1080/07853890.2020.1763449.

[7] sst.dk (2021) ‘tal-og-overvaagning @ www.sst.dk’. Available at: https://www.sst.dk/da/corona/status-for-epidemien/tal-og-overvaagning.

[8] Stasi, C. *et al.* (2020) ‘Treatment for COVID-19: An overvieuw’, (January).

[9] Sterne, J. A. *et al.* (2016) ‘ROBINS-I: A tool for assessing risk of bias in non-randomised studies of interventions’, *BMJ (Online)*, 355, pp. 4–10. doi: 10.1136/bmj.i4919.

[10] Vandenbroucke, J. P. *et al.* (2007) ‘Strengthening the Reporting of Observational Studies in Epidemiology (STROBE): Explanation and elaboration’, *PLoS Medicine*, 4(10), pp. 1628–1654. doi: 10.1371/journal.pmed.0040297.

[11] WHO (2021) ‘Index @ Covid19.Who.Int’, *Https://Covid19.Who.Int/*, p. 1. Available at: https://covid19.who.int/.

[12] Zhang, X. *et al.* (2020) ‘ACE2 and COVID-19 and the resulting ARDS’, *Postgraduate Medical Journal*, pp. 403–407. doi: 10.1136/postgradmedj-2020-137935.
